# Sustainable Design of High-Performance Polyurethanes Using Medium-Chain-Length Polyhydroxyalkanoates

**DOI:** 10.3390/polym18121525

**Published:** 2026-06-18

**Authors:** Jasmina Nikodinovic-Runic, Chebrolu Venkateswara Rao, Maciej Guzik, Malgorzata Zimowska, Dusan Milivojevic, Marijana Ponjavic

**Affiliations:** 1 Institute of Molecular Genetics and Genetic Engineering, University of Belgrade, Vojvode Stepe 444a, 11052 Belgrade, Serbia; jasmina.nikodinovic@imgge.bg.ac.rs (J.N.-R.); chvenkat15@gmail.com (C.V.R.); dusan.milivojevic@imgge.bg.ac.rs (D.M.); 2 Jerzy Haber Institute of Catalysis and Surface Chemistry, Polish Academy of Sciences, Niezapominajek 8, 30-239 Krakow, Poland; maciej.guzik@ikifp.edu.pl (M.G.); nczimows@cyf-kr.edu.pl (M.Z.)

**Keywords:** mcl-polyxydroxyalkanoates, bio-polyurethane, castor oil, sustainable materials, green polyurethanes

## Abstract

The transition toward a circular economy is accelerating the development of high-performance, sustainable polymeric materials derived from renewable resources. Medium-chain-length polyhydroxyalkanoates (mcl-PHAs) represent a versatile class of biodegradable polyesters with inherent flexibility and tunable side-chain chemistry, making them attractive candidates for advanced polymer applications. Here, we report a novel class of bio-based polyurethanes (PUs) incorporating mcl-PHAs as soft segments, marking their first application in polyurethane synthesis and shifting towards greener PU synthesis. Polyurethane networks were prepared using castor oil (CO) and mcl-PHAs as polyols, with hexamethylene diisocyanate (HMDI) as a hard segment. Material properties were systematically tuned by varying the mcl-PHA/CO ratio (100/0 to 0/100), enabling precise control over structure–property relationships. Comprehensive characterization confirmed urethane bond formation and revealed predominantly amorphous materials with tunable thermal and mechanical behavior. Increasing mcl-PHA content enhanced elasticity and influenced phase organization, underscoring its role as a flexible, bio-derived soft segment. The resulting materials exhibited competitive mechanical performance alongside adjustable swelling behavior and morphology. Importantly, in vitro biocompatibility (MRC-5 fibroblasts) and eco-toxicological evaluation (*Caenorhabditis elegans*) confirmed the absence of toxicity. These findings highlight the potential of mcl-PHAs as sustainable building blocks for advanced polyurethane systems.

## 1. Introduction

In recent years, there has been a growing global interest in the development of sustainable and eco-friendly materials to address the pressing challenges posed by the depletion of fossil fuel resources and the accumulation of non-biodegradable plastic waste [1,2,3,4,5,6]. The search for innovative alternatives to conventional plastics has led to significant advancements in the field of biopolymers and biodegradable materials. Among these, polyhydroxyalkanoates (PHAs) have emerged as a promising class of biopolymers due to their remarkable biocompatibility, flexibility, biodegradability, and thermoplastic properties. One particular member of the PHA family is medium-chain-length polyhydroxyalkanoate (mcl-PHA), a versatile and renewable resource for various applications [7,8,9,10,11,12]. Its unique properties, including flexible chain structures make it suitable for blending with other polymers to enhance their properties and biodegradability. Blending mcl-PHA with natural polymers such as starch, lignin, and cellulose derivatives enhances its functionality by providing extended stability, improved elasticity, and reduced oxygen permeability, which are critical for maintaining product shelf life and mechanical resilience [13]. At the same time, mixing with biodegradable synthetic polymers like PLA, PCL, PBAT, and PVA significantly improves biodegradability, processability, and mechanical performance, making the resulting blends more versatile and suitable for large-scale industrial applications [14]. As highly flexible polymers with a low melting point (often below 60 °C), mcl-PHAs are frequently used as property-modifying agents or plasticizers in polymer blends [15]. Their inherent elasticity and flexibility put the focus on mcl-PHAs as a material with great potential in soft tissue engineering (in wound dressings, surgical sutures, cardiac patches, cartilage repair, heart valve substitutes), where mechanical compliance and resilience are essential [16]. Despite these advantageous properties, the incorporation of PHAs, especially mcl-PHAs, into polyurethane networks remains largely unexplored. While PHAs are extensively studied as thermoplastics and biodegradable materials, their role as reactive building blocks in advanced polymer architectures is still underdeveloped [17,18]. This represents a significant opportunity to exploit their intrinsic flexibility and functionality in polyurethane systems.

Polyurethanes are widely used in diverse applications due to their excellent mechanical properties, durability, and chemical resistance [19,20,21,22]. However, conventional polyurethane production is heavily dependent on petroleum-based feedstocks, which are associated with high greenhouse gas emissions and long-term environmental persistence. In this context, the incorporation of bio-based and biodegradable components, such as mcl-PHA and castor oil (CO), offers a promising avenue for the synthesis of sustainable polyurethane films, stressing the potential of bio-PUs to serve as a bridge between high-performance materials and circular economy principles. CO, a versatile vegetable oil, has gained attention as a potential renewable source for industrial applications [23]. Its unique chemical structure offers ester, hydroxyl functionality, and unsaturation [24]. These characteristics make castor oil an attractive feedstock for polyols, and its chemical versatility allows for the production of polyurethane films with tunable mechanical and barrier properties. Over the past few years, CO-based PUs have been intensively investigated, with researchers highlighting their potential as sustainable alternatives to petroleum-derived polymers in various applications [25]. The applicative potential of CO-PU composites reinforced with natural fibers, showing improved mechanical strength and reduced water absorption, is found in packaging due to their durability and moisture resistance [26]. Developing of fast-curing CO-PU coatings demonstrates strong thermal stability, water resistance, and corrosion protection [27]. Towards greener production of PUs, recyclable CO-PU networks, modified with bio-based crosslinkers such as vanillin, were successfully produced, offering both enhanced tensile strength and the ability to undergo multiple recycling cycles, which represents a major advantage for packaging films, which are often single-use and contribute significantly to plastic waste [28]. CO-PU films with the outstanding UV-shielding and visible light-transmitting properties, in combination with remarkable thermal and mechanical properties, were produced employing a three-step synthetic strategy [29].

In this work, we report, for the first time, the use of mcl-PHAs as polyol components in polyurethane synthesis, combined with castor oil to form bio-based PU networks. The presented study represented the first demonstration of mcl-PHAs serving as polyol component with or without integration with castor oil in polyurethane synthesis, establishing bio-based PU networks, advancing the field of sustainable polymer chemistry and opening new pathways for high-performance, biodegradable materials. While our previous study introduced, for the first time, the addition of bacterial biomasses into castor-oil-based PUs as fillers, the present study took a decisive step forward, highlighting the great potential of bio-based mcl-PHA as a polyol component in PU synthesis moving toward greener PU production. By systematically varying the CO/mcl-PHA ratio, we demonstrated that the structure–property relationships of the resulting materials can be effectively tuned without chemical modification. The synthesized PUs exhibit high crosslinking efficiency, a predominantly amorphous structure, and composition-dependent thermal and mechanical behavior, with mcl-PHA incorporation enhancing flexibility while maintaining competitive performance. Importantly, the materials display low swelling in polar media, tunable surface wettability, and confirmed biocompatibility and environmental safety, a critical point in advancing bio-based polymers toward industrial adoption.

## 2. Materials and Methods

### 2.1. Materials

Virgin castor oil was purchased from Carl Roth (Karlsruhe, Germany), hexamethylenediisocyanate (HMDI) from Sigma Aldrich (purity ≥ 99.0%, Munich, Germany), and ethyl acetate from Merck (HPLC-grade, purity ≥ 99.5%, Darmstadt, Germany), while chloroform, acetone (HPLC-grade, purity ≥ 99.5%), N,N-Dimethylformamide, DMF (99.8%, anhydrous, Sigma Aldrich, St. Louis, MO, USA), and dimethyl sulfoxide, DMSO (Fisher Scientific, Darmstadt, Germany) were supplied by Fisher Chemicals (Geel, Belgium). All chemicals were used without additional purification. For experiments with *Caenorhabditis elegans*, selected chemicals were used: peptone, calcium chloride, CaCl_2_, potassium phosphate buffer, potassium hydrogen phosphate, KH_2_PO_4_, sodium hydrogen phosphate, Na_2_HPO_4_, sodium chloride, NaCl, magnesium sulfate, MgSO_4_, LB broth and cholesterol, all purchased from Sigma-Aldrich (Saint Louis, USA). mcl-PHA (*M*_n_ = 11,800 Da, *M*_w_ = 15,000 Da, PDI = 1.2) was produced by *Pseudomonas putida* CA-3 (NCIMB 41162; National Collection of Industrial, Food and Marine Bacteria) fermentation from canola-oil-derived fatty acids at 30 °C [30].

### 2.2. Synthesis of PUs

The new PU materials were synthesized using the solvent casting method according to the procedure reported in our previous paper [31]. In total, 3.0 g of polyol components (mcl-PHA (*n*_polymer_ = 1.0 g/*M*_n_, *n*_polymer_ ≈ 8.47 × 10^−5^ mol) or castor oil–hydroxyl 167.0 mg KOH/g, or their combination in various ratios) and 3.15 g (3.0 mL) of hexamethylene diisocyanate (HMDI) were mixed in a 250 mL flask equipped with a condenser and refluxed in 30 mL of ethyl acetate as solvent. The reaction mixture was heated over 24 h at 70 °C in an inert atmosphere with continuous stirring (200 rpm). After the predetermined reaction period, the reaction mixture was cast onto the Teflon mold (diameter 10 cm, height 2 cm) and allowed to evaporate at room temperature (23–25 °C) in a fume hood, resulting in mcl-PHA-based polyurethane films. The polyol component composition was varied by adjusting the starting reaction mixture to castor oil–mcl-PHA (C-P) ratios of 20/80, 50/50 and 80/20, calculated to the amount of 3.0 g of polyol. The polyurethanes synthesized by using neat castor oil and mcl-PHA were labeled as C-P 100/0 and C-P 0/100, respectively. The prepared PUs films were immersed in chloroform for 24 h to remove the excess of the potentially unreacted HMDI, dried till constant mass (dried in a vacuum oven and kept in a desiccator) and subjected to characterization.

### 2.3. Characterization of mcl-PHA PUs

The chemical structure of the polyols used for PU synthesis (castor oil and mcl-PHA) was verified using nuclear magnetic resonance (^1^H NMR) spectroscopy. All spectra were obtained on a Bruker Ascend 400 instrument, operating at 400 MHz. Deuterated chloroform (CDCl_3_) served as the solvent, with tetramethylsilane (TMS) used as the internal standard.

Fourier-transform infrared spectroscopy (ATR-FTIR) analysis was applied to confirm urethane linkage formation in the mcl-PHA-based PU films. The films were analyzed using an IR-affinity spectrophotometer (Thermo Fisher Scientific, NICOLET iS10, Waltham, MA, USA) in an attenuated total reflection (ATR) mode, in the predefined wavenumber range of 4000–400 cm^−1^, at room temperature, with a resolution of 4 cm^−1^, and with a fixed number of scans at 32.

For the water uptake tests, the PU films were cut into rectangular shapes of 1 × 1 cm^2^, placed in glass vials, and 10 mL of deionized water was added. The immersed films were incubated at 37 °C, and the water uptake was measured after the predetermined time (1, 3, 5, 7, 10, 14 days, respectively). The excess of water was removed using a filter paper, and the water uptake was estimated by applying Equation (1) [29]:

(1) $$Water\;uptake\;\left(\%\right)=\;\frac{w_{1}-w_{0}}{w_{0}}\;\times 100\;\%$$

where *w*_0_ corresponds to the initial mass of film and *w*_1_ is the mass of PU films after immersion in water. All the measurements were done in duplicate and the final values were presented as an average value ± SD.

The gel fraction and swelling ratio of the prepared PU films were evaluated by their immersion in chloroform. Each PU film was cut into a rectangular shape (1 × 1 cm^2^), placed in 15 mL sealed vials and incubated in 10 mL of chloroform at room temperature (25 °C) for three days. After a predetermined period of time, the excess of solvent was eliminated with filter paper and the swelling ration was calculated according to Equation (2) [28]:

(2) $$Swelling\;ratio\;\left(\%\right)=\;\frac{w_{1}-w_{0}}{w_{0}}\;\times 100\%\;$$

where *w*_0_ is the initial mass of bio-PU film, and *w*_1_ is the mass of the PU film after immersion in chloroform.

In addition, the PU films were dried in the vacuum oven at 70 °C for 24 h, and the mass of dried samples was weighted (*w*_2_) in order to estimate the gel fraction by using Equation (3):

(3) $$Gel\;fraction\;\left(\%\right)=\;\frac{w_{2}}{w_{0}}\;\times 100\%$$

Furthermore, solvents of different polarity, including water, dimethylformamide (DMF) and dimethyl sulfoxide (DMSO), were also used for the evaluation of the swelling ability of tested PU films following the same procedure applied for chloroform as the solvent. All tests were performed in duplicate and the final values are shown as an average values ± SDs.

The water contact angle (WCA) was measured using the Kruss Drop Shape Analyzer DSA100M (Krüss GmbH, Hamburg, Germany) equipped with an optical microscope and a digital camera (20 fps) capable of quickly capturing images. The recorder images were further evaluated using a digital-image-processing algorithm to calculate the droplet’s contact angle applying tangents or Laplace–Young approximations. The samples were placed in the test chamber of the controlled testing conditions, at a temperature of 22 ±  0.3 °C and humidity of 30 ± 5%. For each species, at least five successive measurements were done and the average values ± SD are presented.

#### Differential Scanning Calorimetry Analysis (DSC)

Differential scanning calorimetry analysis (DSC) was performed using a Shimadzu—DSC-60 Plus under a nitrogen atmosphere, with a gas flow of 50 mL/min, in a temperature range from −50 to 250 °C, with a heating rate of 10 °C/min. The mass of the test PU sample was 5.0 ± 0.5 mg. The observed thermograms were further analyzed (melting temperatures, *T*_m_, glass transition temperatures, *T*_g_) by Origin Pro 9.0 software.

Thermogravimetric (TG) measurements were done using a TA Instruments SDT Q600 (New Castle, DE, USA) by applying the following conditions: a temperature range from 25 °C to 800 °C, a heating rate of 20 °C/min, in a nitrogen atmosphere with a flow of 20 mL/min. The analyzed PU films mass were in the range of 5–7 mg.

The mechanical properties of the PU films were assessed using a Shimadzu Autograph AGS-X servo-hydraulic testing machine (Kyoto, Japan) fitted with a 1 kN load cell, following ASTM D638 standards [32] at ambient temperature. Rectangular specimens (4.0 × 1.5 cm^2^) were tested at a testing speed of 2 mm/min, with five replicates per sample. The average values of tensile strength at break, elongation at break, and Young’s modulus (±standard deviation) were calculated. Young’s modulus was determined by the TRAPEZIUM X-V software from the linear region of the stress–strain curve.

Wide angle X-ray diffraction (XRD) spectra were collected with the X’Pert PRO (PANalytical B.V., Almelo, The Netherlands) diffractometer operated at 40 kV and 30 mA by using Ni-filtered Cu Kα radiation. PU films were recorded in the range of 2*θ* = 5–50°, with a step size of 0.02°.

An inspection of the PU surface morphology was done using scanning electron microscopy (SEM, JEOL JSM-6390LV JEOL USA Inc., Peabody, MA, USA) by applying an accelerating voltage of 20 kV. Prior to recording, PU films were attached to a double-sided carbon adhesive tape on top of an aluminum stud and sputtered by a thin layer of Au.

The optical properties of the prepared mcl-PHA PU films, including transparency, were analyzed by measuring the transmittance percentage using an ultraviolet–visible (UV-Vis) spectrophotometer (UV-1800, Shimadzu, Kyoto, Japan). For these measurements, the films were cut into rectangular pieces (1 × 4 cm^2^) and placed directly on the side of spectrophotometer cells, applying air as a reference in the wavelength range of 200–800 nm.

### 2.4. Cytotoxicity Assessment

Cytotoxicity was evaluated using a human fibroblast line (MRC-5) obtained from the ATCC culture collection, following the standard MTT assay [33]. Test material extracts were prepared by incubating film samples (10 mg/mL) in RPMI-1640 medium for 72 h at 37 °C. The obtained extracts were used for the cell treatment of different concentrations, 100%, 50%, 25%, and 12.5% (*v*/*v*), and treated MRC-5 fibroblasts were allowed to grow for 48 h. Cell proliferation was assessed using the MTT reduction assay, with absorbance measured at 540 nm and 670 nm on an Epoch multiplate reader (BioTek Instruments, Inc., Winooski, VT, USA). Standard culture medium and untreated cells served as controls, while the results were expressed as a percentage relative to the control, which was set to 100%.

*Caenorhabditis elegans* AU37 (glp-4; sek-1) was propagated under standard conditions, synchronized by hypochlorite bleaching, and cultured on nematode growth medium (2.5 g of peptone, 3.0 g of NaCl, 20.0 g of agar, 1 mL of 1M CaCl_2_, 25 mL of KPO_4_ buffer (pH 6), 1 mL of 1M MgSO_4_·7H_2_O and 1 mL of cholesterol (conc. 5 mg/mL)) using *E. coli* OP50 as a food source, as described previously [34]. The *C. elegans* survival assay was carried out as described previously with appropriate adaptations [35,36]. In brief, synchronized worms (L4 stage) were suspended in a medium containing 95% M9 buffer (3.0 g of KH_2_PO_4_, 6.0 g of Na_2_HPO_4_, 5.0 g of NaCl, and 1.0 mL of 1M MgSO_4_ in 1 L of water), 5% LB broth, and 10 μg of cholesterol per mL. The experiment was carried out in 96-well flat-bottomed microtiter plates (Sarstedt, Germany) in a final volume of 100 μL per well. Initially, materials were incubated in a medium composed of 95% M9 buffer, 5% LB, and 10 μg of cholesterol for 48 h at 25 °C, with a material-to-medium ratio of 100 g/100 mL. In addition, 50 μL of material extracts was transferred to a well in a 96-well plate, where 50 μL of a nematode suspension (25–35 nematodes) was added. Subsequently, the plates were incubated at 25 °C for 2 days. The fraction of dead worms was determined after 48 h by counting the number of dead worms and the total number of worms in each well, using a stereomicroscope (SZX10 Olympus, Boston, MA, USA). The materials were tested at least three times in each assay, and each assay was repeated at least two times (*n* ≥ 6). As a negative control experiment, nematodes were exposed to a standard medium.

### 2.5. Statistical Analysis

GraphPad Prism 9.4.1 software (GraphPad Prism 9.4.1, La Jolla, CA, USA) was used to perform a one-way analysis of variance (ANOVA), followed by Tukey’s multiple-comparison test. The final results are presented as mean values ± standard deviation (SD), considering a statistically significant difference at *p* ≤ 0.05.

## 3. Results

### 3.1. Synthesis of mcl-PHA-Based PUs

**Figure 1:**
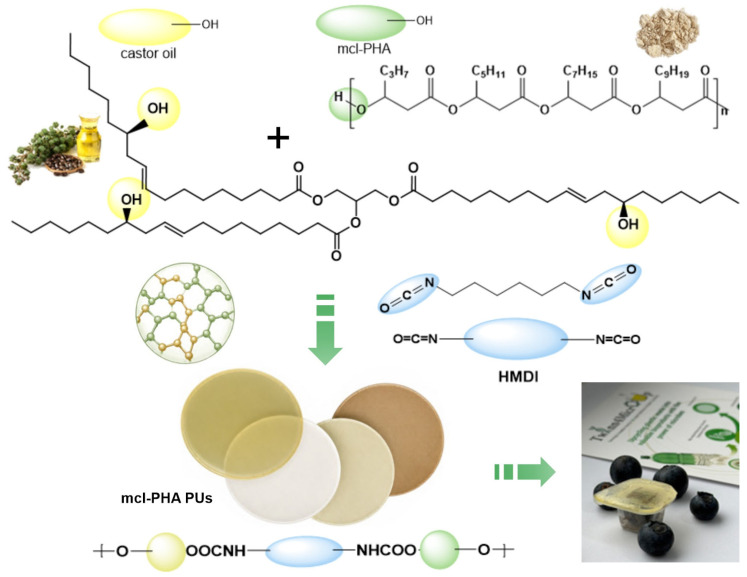
Conceptualization of this study: obtaining new mcl-PHA-based PUs.

The synthesis of high-performance bio-polyurethanes (mcl-PHA PUs) demonstrated that the use of mcl-PHAs as a renewable polyol component offers the sustainable solution for the development of new PU materials. Prior to the PU synthesis, the chemical structure of the polyol components (CO and mcl-PHA) was confirmed by NMR spectroscopy (Figure S1) and the obtained results were consistent with the expected chemical structure and in agreement with the characteristic peaks reported in the literature [28,37]. Five PU films of varied polyol composition were prepared by adjusting the ratio of castor oil to mcl-PHA (Figure 1), while the amount of added crosslinker, HMDI, was constant (Table 1). The obtained yellowish PUs films were of a high yields and similar for all samples (~85%), indicating that the CO/mcl-PHA ratio had no prominent influence on the overall reaction conversion efficiency.

Film thickness varied with polyol composition (Table 1). The PU derived solely from castor oil (C-P 100/0) exhibited a thickness of 1.18 mm, whereas the lowest thickness was observed for the mcl-PHA-based sample (C-P 0/100), measuring 0.71 mm, amounting to a 1.6-fold difference. Interestingly, the PU prepared with an equimolar polyol ratio (C-P 50/50) displayed the greatest thickness (1.33 mm), suggesting a possible synergistic effect of the combined polyols on network formation and crosslinking density.

The water contact angle values listed in Table 1 indicate variations in surface wettability of mcl-PHA PU films, which can be attributed to differences in polyol segment composition. The C-P 100/0 PU films with castor oil had an average of 83.5°, suggesting a hydrophilic behavior, while the PU containing mcl-PHA as a polyol component indicated quite high values of WCA, above 116°. PU films prepared with an equal ratio of castor oil to mcl-PHA demonstrated the highest hydrophobicity, achieving a WCA of 122°. Notably, further increases in the amount of inherently hydrophobic mcl-PHA did not result in higher WCA values.

#### 3.1.1. ATR-FTIR Analysis

**Figure 2:**
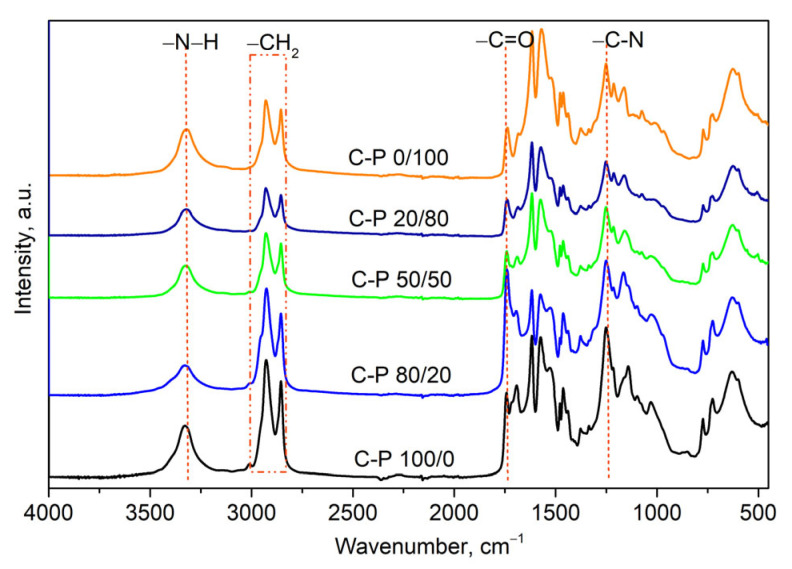
FTIR spectra of the synthetized bioPUs.

The FTIR spectrum of new PU films served as confirmation of their structure and the identification of urethane bond formation. The presented spectra demonstrated the characteristic peak ranged from 3325 cm^−1^ to 3000 cm^−1^, which corresponded to N-H vibrations, while the peak at 1613 cm^−1^ corresponded to N-H bending vibrations (Figure 2). The appearance of characteristic peak at wavenumber 1747 cm^−1^ was attributed to carbonyl -C=O stretching vibrations, while the strong peak located at 1250 cm^−1^ is a result of the stretching -C-N. The recorded spectra undoubtedly confirmed the successful formation of urethane linkage (-NHCOO-) in the synthesized PUs [20]. Apart from the characteristic peaks inherent to urethane bond, the absorbance band detected in the range of 2852–2928 cm^−1^ indicated the asymmetric and symmetric stretched vibrations of aliphatic -CH_2_ [28].

To determine the hydrogen interactions of the PU film components, the wavenumber regions from 1600 to 1800 cm^−1^ were deconvoluted into three peak components: of esters at 1740 cm^−1^, free C-O at 1720 cm^−1^ and H-bonded C-O at 1690 cm^−1^ (Figure S2) [38]. Specifically, a lower wavenumber in the -C=O stretching vibration indicates stronger hydrogen bonding [39].

The deconvolution results of the carbonyl of PU samples are shown in Table S1, obtained by applying Gaussian curve fitting. The compositional ratio of bonded C-O for C-P 100/0 was 69.2%, the highest in the series because of the established hydrogen bonds with urethane groups in PU. Also, the free -C-O component in this sample was the lowest in all PUs, confirming stronger hydrogen bonding. When mcl-PHA was used as single polyol component (C-P 0/100), the compositional ratio of free -C-O was high (18%), while the ratio of the bonded was 57.5%, indicating that the hydrogen bonds were rather weak. However, blending of CO and mcl-PHA as polyol components resulted in values of the compositional ratio close to those of the C-P 100/0 film, suggesting the availability of the present groups to establish hydrogen bonds with urethane linkage. Both crosslinking and the established hydrogen bonds dictated the physicochemical properties of the obtained PU materials.

#### 3.1.2. Swelling Tests

**Figure 3:**
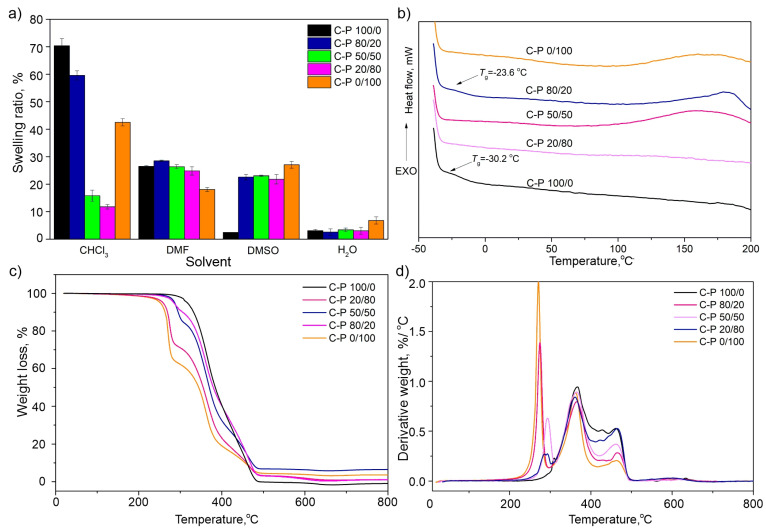
(**a**) Swelling properties of mcl-PUs in different solvents, (**b**) DSC analysis of the obtained mcl-PUs, (**c**) TGA and (**d**) DTGA curves of the investigated PUs.

The swelling behavior of PU films was evaluated in solvents of varying polarity, including chloroform, water, DMF, and DMSO (Figure 3a). Swelling in chloroform was additionally used to determine the gel fraction, representing the insoluble portion of the polymer network. The gel fraction, indicative of crosslinking density, varied with polyol composition and the availability of reactive -OH groups in the polyols (Table 1). The highest gel fraction was observed for the CO-rich sample (C-P 100/0, ~99.8%), while the lowest value was obtained for C-P 20/80 (~87.8%), suggesting reduced crosslinking density. All samples exhibited pronounced swelling in chloroform, with values strongly dependent on composition. The C-P 100/0 sample showed the highest swelling (~70%), followed by C-P 80/20 (~60%) and C-P 0/100 (~40%), whereas samples with a lower CO content exhibited limited swelling (≤10%). In contrast, swelling in DMF and DMSO was moderate (20–30%) and relatively consistent across compositions. Due to the hydrophobic nature of mcl-PHA-based PUs, swelling in water as a highly polar solvent was minimal (<5%). These results confirm that both solvent polarity and polyol composition significantly influence network structure and solvent uptake behavior.

As mentioned, the water contact angle values highlighted the differences in surface wettability among mcl-PU films attributable to the variety in polyol segment composition (Table 1). Water resistance, a highly desirable property for many PU applications, was additionally confirmed by water uptake experiments where the tested PU films showed a low water uptake capacity, below 1 wt%. The water uptake ability of mcl-PUs as a function of time during the immersion process is shown in Figure S1b.

#### 3.1.3. Thermal Properties of the mcl-PHA PUs

The DSC thermograms of the mcl-PUs are presented in Figure 3b, with the corresponding thermal parameters summarized in Table 2. The glass transition temperature (*T_g_*) was detected only for the CO-rich samples, namely C-P 100/0 (30.2 °C) and C-P 80/20 (23.6 °C), indicating that castor-oil-derived segments dominate the soft-phase dynamics in these systems. The decrease in *T_g_* with increasing mcl-PHA content suggests enhanced chain mobility and reduced segmental interactions, attributable to the flexible aliphatic side chains of mcl-PHAs. For compositions with higher mcl-PHA fractions, *T_g_* was not clearly observed, likely due to increased phase mixing or broadened transitions arising from heterogeneous segment distribution.

No distinct melting or crystallization transitions were detected within the −70 °C to 200 °C range, indicating predominantly amorphous structures with suppressed crystallinity. These findings suggested that the incorporation of mcl-PHAs promotes amorphous, elastomeric networks with enhanced flexibility, which is further supported by XRD analysis.

The results determined by TGA/DTGA (Figure 3c,d and Table 2) provided insight into the influence of polyol component composition (C-P ratio) on processability and thermal stability, which are crucial for PU applications. The characteristic temperatures indicating 5%, 50% and 90% of weight loss temperatures (*T*_5%_, *T*_50%_, and *T*_90%_, respectively) denoted in Table 2 confirmed high thermal stability up to the temperatures range from 250 to 316 °C, when 5% of weight loss was observed. For the PU film C-P 100/0, the highest onset degradation (*T*_5%_ = 316 °C) and remarkable thermal stability were observed, contrary to PU C-P 0/100, with mcl-PCL as the polyol, which had the lowest *T*_5%_ = 250 °C, indicative of desirable but the lowest thermal stability in the synthesized series.

DTGA analysis also revealed composition-dependent degradation behavior. The CO-based PU exhibited a two-step degradation process at *T*_max_ ~ 365 °C and 460 °C, whereas mcl-PHA-based PU showed a three-step profile *T*_max_ ~ 270, 363, 465 °C. While the decomposition of CO-based PU referred to the decomposition of the urethane bonds and aliphatic chains in the first stage, the second degradation phase occurred due to the oxidative degradation of isocyanates, the breakdown of crosslinking linkages. The additional low-temperature degradation step in mcl-PHA-rich samples is attributed to the presence of less thermally stable aliphatic side chains. Increasing mcl-PHA content intensified this initial degradation stage, while its reduction shifted the onset to higher temperatures and improved overall thermal stability, indicating the stabilizing effect of CO-derived segments.

#### 3.1.4. XRD Analysis

**Figure 4:**
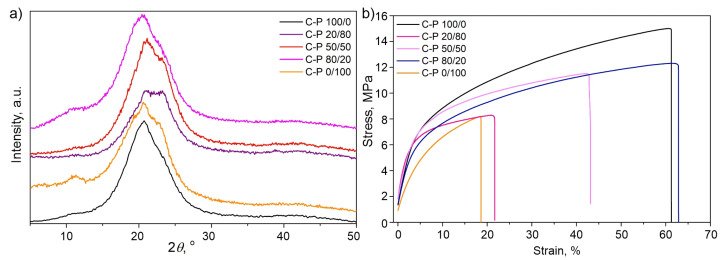
(**a**) The XRD diffractograms and (**b**) stress–strain graphs of synthesized mcl-PHA PUs.

XRD analysis was performed to investigate the crystalline structure of the synthesized mcl-PHA-based polyurethanes (Figure 4a). The XRD pattern showed a broad diffraction peak detected for all PU films, at 2*θ* values of ≈20° indicating an amorphous structure of the samples with no distinct crystalline peaks observed all over the diffraction spectra. However, a small, less intense, and relatively broad crystalline peak, with the characteristic crystalline reflections for mcl-PHAs, appeared around 2*θ* = 12.0° in the diffractogram of mcl-PHA-rich PU. This is in a line with previous studies investigating castor-oil-based PUs [40].

#### 3.1.5. Mechanical Tests

Mechanical properties are critical for PU films as they determine durability, flexibility, and suitability for applications in both biomedical and industrial fields. Tailoring the composition of soft and hard segments provides an effective strategy for tuning tensile strength, elasticity, and degradation behavior, enabling a balance between functionality and sustainability.

The mechanical performance of the synthesized PU films (Figure 4b, Table 3) strongly depended on polyol composition. The CO-based PU (C-P 100/0) exhibited the highest tensile strength, elongation at break, and Young’s modulus (14.2 MPa, 54.2%, and 171.6 MPa, respectively). In contrast, the mcl-PHA-rich sample (C-P 0/100) showed the lowest values (7.49 MPa, 18%, and 99.1 MPa), reflecting reduced mechanical integrity. Notably, the C-P 80/20 formulation displayed slightly higher elongation at break (59.6%) compared to the CO-based sample while maintaining a comparable modulus (169.3 MPa), suggesting improved flexibility without significant loss of stiffness. Overall, increasing mcl-PHA content led to a gradual reduction in mechanical strength, highlighting the key role of polyol composition in governing material performance.

#### 3.1.6. SEM Analysis

**Figure 5:**
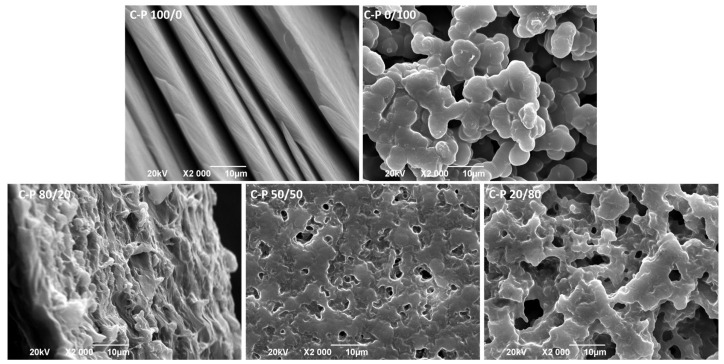
SEM images of the synthesized mcl-PHA PUs (magnification of 2000×).

The morphology of the mcl-PUs was assessed by SEM analysis and the captured images are shown in Figure 5. The SEM analysis of the C-P 100/0 PU film showed a smooth homogenous surface morphology commonly observed for castor-oil-based PUs [41], while C-P 0/100 indicated a more heterogeneous surface with clearly visible polymer agglomerates and phase-separated segments. Increasing the mcl-PHA content reduced miscibility with CO, leading to rougher, more heterogeneous surfaces because longer PHA chains interfered with uniform packing, explaining the irregular textures.

#### 3.1.7. Safety Assessment of the mcl-PHA PUs

**Figure 6:**
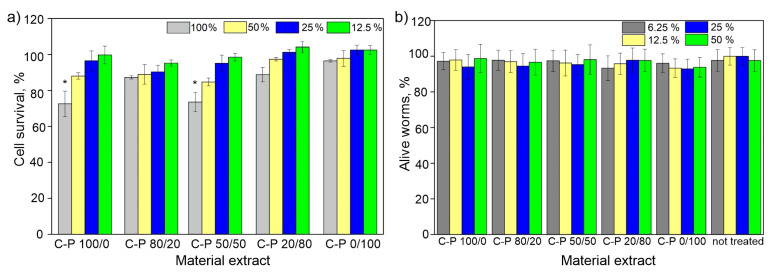
Safety assessment of mcl-PHA PUs using: (**a**) MRC-5 cells and (**b**) *C. elegans*; Statistically significant differences relative to the control are indicated by an asterisk (* *p* ≤ 0.05).

Assessment of material toxicity is essential to ensure safe biological interactions and reliable performance, particularly for applications involving human and food contact. Therefore, the cytotoxicity and eco-toxicity of mcl-PHA-based PU extracts were evaluated using human fibroblasts (MRC-5) and the model organism *C. elegans* (Figure 6). As shown in Figure 6a, extracts from C-P 100/0, C-P 20/80, and C-P 50/50 films induced a dose-dependent decrease in fibroblast proliferation at higher concentrations (inhibiting cell proliferation of up to 30%), whereas lower dilutions showed minimal or no effect. Interestingly, C-P 80/20 and C-P 0/100 did not induce any cytotoxicity. Importantly, cell viability remained above the 70–80% threshold in all cases, indicating that the materials can be classified as non-cytotoxic under the tested conditions. Consistently, exposure of *C. elegans* to PU extracts did not result in observable toxic effects (Figure 6b). Together, these findings confirm the biocompatible and environmentally safe profile of the synthesized mcl-PHA-based polyurethanes, supporting their potential for applications requiring safe biological and ecological interactions.

### 3.2. Transparency of the mcl-PHA PU Films and Their Potential Application

**Figure 7:**
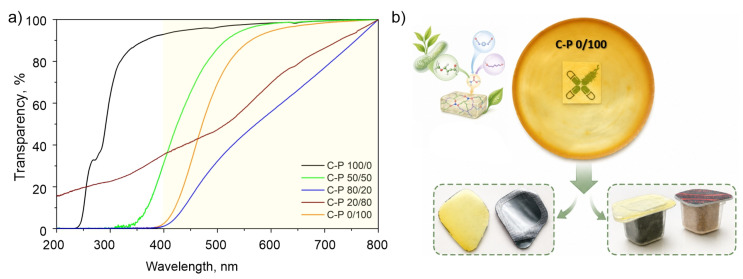
(**a**) Transparency properties and (**b**) proof of concept—potential application of new mcl-PHA PUs.

The optical properties of the new PU films were defined using the transmittance tests of the mcl-PHA PUs (Figure 7a). The recorded UV–Vis spectra in transparency mode revealed a clear dependence of transparency on the relative composition of the two polyol components. The high transparency across the whole visible region (400 to 800 nm) was achieved in CO-rich PUs, with the C-P 100/0 film being fully transparent (T% above 90% over the visible range), while blending with mcl-PHA resulted in less transparent PU films due to the yellowish color of mcl-PHA itself. Films with mixed ratios (C-P 80/20 and C-P 20/80) showed moderate transparency, while the mcl-PHA-rich film, C-P 0/100, indicated T% above 80% in the range up to 500 nm. The differences between PU films confirmed the critical role of the polyol ratio in tuning optical properties. Focusing on balancing the desirable mechanical, thermal properties of new mcl-PHA PUs with optical properties, absence of toxicity and sustainability, the C-P 0/100 PU film was selected for the proof of concept in packaging applications.

Figure 7b demonstrates a proof-of-concept application of mcl-PHA-based polyurethanes as covering foils. The transparent films produced from the C-P 0/100 PU formulation exhibited not only high transparency but also notable mechanical versatility as they could be readily cut, molded, and shaped into practical forms such as sheets and flexible films. This visual evidence further supports the potential of mcl-PHA-based PUs for packaging and protective applications, where a combination of transparency, flexibility, processability, and sustainability is highly desirable.

## 4. Discussion

The development of mcl-PHA-based polyurethanes represents a significant step toward sustainable, high-performance materials by integrating renewable feedstocks with tunable physicochemical properties. The synthesized mcl-PHA PUs, prepared with varying CO/mcl-PHA ratios, were obtained in consistently high yields (83–86%), indicating that the reaction efficiency was largely unaffected by composition. High gel fractions (88–99%) further confirmed effective network formation, with CO-rich systems exhibiting higher crosslinking density due to the greater availability of reactive hydroxyl groups. In contrast, increasing mcl-PHA content reduced gel fraction, reflecting lower functionality and a less tightly crosslinked network. This trend is consistent with previous reports on vegetable-oil-based PUs, where higher hydroxyl functionality leads to increased crosslink density and network integrity [42]. However, the present system uniquely demonstrates that incorporation of mcl-PHAs enables modulation of crosslinking without compromising synthesis efficiency, which remains less explored in bio-based PU design.

FTIR analysis confirmed urethane formation and revealed that both covalent crosslinks and hydrogen bonding contribute to network stability. Deconvolution of the carbonyl region demonstrated that CO-rich systems exhibited stronger hydrogen bonding, while mcl-PHA-rich compositions showed increased free carbonyl content, indicating weaker intermolecular interactions. These differences directly influence macroscopic properties, highlighting the interplay between chemical structure and physical behavior. Similar hydrogen-bonding effects have been widely reported in conventional segmented polyurethanes [39,43], where hard segment interactions govern mechanical performance. In contrast, the present results emphasize how bio-derived soft segments such as mcl-PHAs can actively modulate these interactions, offering an additional design parameter for tailoring material properties.

Swelling behavior further reflected this structure–property relationship. All samples showed the highest swelling in chloroform, with uptake decreasing as CO content decreased, consistent with reduced crosslink density and altered segment interactions. Moderate swelling in DMF and DMSO and minimal water uptake (<5%) confirmed the predominantly hydrophobic nature of the materials. Despite the obvious difference in water uptake between PUs films of different polyol composition, water absorption lower than 1% for 14 days was considered insignificant and the potential differences in the series were investigated by WCA analysis. Moreover, the water uptake increases with the decrease in crosslink density [44]. The obtained results were correlated with swelling tests in water, and generally hydrophobic behavior was observed. Castor-oil-based PUs generally exhibit good solvent resistance due to the high crosslinking density provided by the triglyceride structure of castor oil [45].

Water contact angle analysis supported the findings of swelling behavior, demonstrating that surface wettability is not a linear function of composition but can be effectively tuned by adjusting the CO/mcl-PHA ratio, enabling application-specific surface design. Recognizing a material surface with a water contact angle of 90° or higher as non-wetting [46], three PU films, C-P 0/100, C-P 20/80, C-P 80/20, can be described as hydrophobic, where water spreads slowly. A WCA value lower than 90°, measured for the PU C-P 100/0 and C-P 50/50, indicated a hydrophilic surface. The measured WCA results revealed that hydrophobicity is not a linear function of mcl-PHA content, offering a tunable platform for tailoring PU films to specific applications in terms of wettability. Despite the possibility of enhanced water resistance performance of castor-oil-based PUs being achieved by chemical modification [47], this study implied that simply by varying polyol composition, without chemical modifications, control of surface properties could be achieved.

Thermal analysis revealed predominantly amorphous structures, with *T*_g_ values observed only in CO-rich systems, reflecting restricted segmental mobility [48]. Increasing mcl-PHA content enhanced chain flexibility and suppressed crystallinity, as confirmed by XRD. Thermal degradation behavior was also composition-dependent: CO-rich PUs exhibited higher thermal stability, while mcl-PHA incorporation introduced an additional low-temperature degradation step associated with aliphatic side chains. The thermal decomposition of the CO-based PUs preferentially happens through the two-stage mechanism: the first one is related to the thermal degradation of the hard segment of low thermal stability of the urethane groups [27], whereas the second degradation step corresponds to the decomposition of soft segments [49]. It is known that mcl-PHAs degrade mainly between 200 and 300 °C, with an onset around 250 °C; it was evident that the first degradation maximum in DTGA was a result of the decomposition of the mcl-PHA segment [50]. The thermal degradation of the mcl-PHA PUs further proceeded at 300 to 400 °C due to the decomposition of the urethane bonds and aliphatic chains, as well as due to the breaking of the ester bond components in castor oil; the first stage occurred in the temperature range of 300 to 400 °C due to the decomposition of the urethane bonds and aliphatic chains [28,51]. The last degradation phase occurred mostly through the oxidative degradation of isocyanates, the breakdown of crosslinking linkages, and other chemical bonds [52].

XRD analysis of mcl-PHA typically revealed a predominantly amorphous, elastomeric structure with a broad amorphous peak around 2*θ* ≈ 20° and hardly detectable small intensity peaks reflecting the crystalline domains observed at 2*θ* ≈ 11° and 2*θ* ≈ 22° [53,54]. CO-based PUs are mostly recognized as rather amorphous with a broad, amorphous halo diffraction peak [39]. Despite the differences in the visual appearance of the XRD spectra of C-P 20/80 and C-P 50/50, with shoulder-like small peaks at ~23°, the synthesized PU films were preferentially considered as amorphous, with not-very-expressed reflectiveness of the polyol composition on the XRD patterns.

The mechanical properties followed a similar trend, with CO-rich samples showing higher strength and stiffness, while mcl-PHA incorporation enhanced flexibility but reduced mechanical integrity at high loadings. CO-based PUs with shape-memory behavior had tensile strength similar to that of mcl-PHA PUs [55]. However, the green PU thermosets with the polyol components derived from hemicellulose sugars with variability in composition appeared to have reduced elongation at break and tensile strength for some PU samples [56]. Morphological analysis (SEM) revealed a transition from smooth, homogeneous surfaces in CO-rich systems to more heterogeneous and phase-separated structures in mcl-PHA-rich materials, consistent with reduced compatibility and increased segmental heterogeneity. This behavior parallels that observed in other segmented PU systems [20], where increased soft-segment content typically enhances elongation but reduces tensile strength. However, the retention of moderate mechanical performance even at higher mcl-PHA content highlights the potential of these bio-derived segments to achieve a balance between elasticity and strength not always attainable in conventional systems.

Cytotoxicity and eco-toxicity assessments confirmed the absence of adverse effects, supporting the biocompatibility and environmental safety of the developed materials. Comparable findings have been reported for polyhydroxyalkanoates, where degradation products modulate cellular metabolism without eliciting acute toxicity [57,58].

Castor-oil-based PU films are generally characterized by high optical transparency, often exhibiting transmittance values in the range of 90–98% in the visible region [59]. These sustainable bio-based films achieve excellent clarity due to controlled microphase separation between the hard and soft segments, making them ideal for protective coatings, electronics, and optoelectronic applications [60]. By linking transparency data with the practical demonstration of molded foils, the proof of concept conveyed that mcl-PHA PUs could move beyond laboratory characterization into real-world uses. Their ability to form transparent and moldable films highlighted their potential as eco-friendly alternatives to petroleum-based plastics in packaging, biomedical coverings, and other applications where optical clarity and non-toxicity are both valuable. Together, these results demonstrate that mcl-PHAs are promising building blocks for advanced polyurethane systems, enabling precise tuning of structure–property relationships while maintaining sustainability and safety.

## 5. Conclusions

This work demonstrates, for the first time, the successful incorporation of medium-chain-length polyhydroxyalkanoates (mcl-PHAs) as polyol components in polyurethane synthesis, establishing a new route toward fully bio-based materials. By systematically adjusting the castor oil (CO)/mcl-PHA ratio, the structure–property relationships of the resulting PUs were effectively controlled without the need for chemical modification. This compositional strategy enabled precise tuning of crosslinking density, thermal behavior, mechanical performance, and surface properties.

The synthesized materials exhibited competitive mechanical properties compared to those of conventional CO-based PUs while offering enhanced flexibility and tunable functionality. High gel fractions, controlled swelling behavior, and predominantly amorphous structures further highlight the versatility of these systems. Importantly, cytotoxicity and ecotoxicity assessments confirmed the absence of adverse effects, supporting their suitability for applications requiring both performance and safety.

Overall, mcl-PHAs emerge as promising and underutilized building blocks for advanced polyurethane design. The ability to tailor properties through simple compositional variation provides a scalable and sustainable platform for next-generation materials. Future work should explore non-isocyanate polyurethane routes and biodegradation pathways to further enhance environmental compatibility and expand application potential.

## Figures and Tables

**Table 1:** The composition of reaction mixture in feed, the obtained yields, gel fraction, water contact angle and the thickness of the synthesized PUs.

Sample	Castor Oil, g	mcl-PHA, g	HMDI, g	Yield, %	Thickness, mm	Gel Fraction (%)	Water Contact Angle, °
C-P 100/0	3.0	0.0	3.15	85.9	1.18 ± 0.02	99.8 ± 0.23	87.3 ± 1.4
C-P 80/20	2.4	0.6		85.4	0.90 ± 0.07	98.3 ± 0.01	122.2 ± 8.7
C-P 50/50	1.5	1.5		85.0	1.33 ± 0.04	91.6 ± 0.19	93.2 ± 6.0
C-P 20/80	0.6	2.4		83.4	1.14 ± 0.11	87.8 ± 0.47	116.9 ± 1.1
C-P 0/100	0.0	3.0		85.2	0.71 ± 0.04	95.7 ± 0.04	117.5 ± 5.9

**Table 2:** The DSC and TGA/DTG analysis results of the mcl-PHA-based PUs.

Sample	T_g_ *, °C	T_5%_, °C	T_50%_, °C	T_90%_, °C	T_max_, °C	Residual Weight, %
C-P 100/0	−30.2	316.1	385.5	461.1	365.4/460.7	-
C-P 80/20	−23.6	283.5	343.6	469.2	274.7/366.0/464.7	1.00
C-P 50/50	n.d. **	285.5	379.4	472.8	292.6/365.4/462.7	6.8
C-P 20/80	n.d. **	259.3	370.5	458.8	288.7/360.7/463.6	1.00
C-P 0/100	n.d. **	250.8	355.6	454.7	270.0/362.7/464.7	3.54

* by DSC analysis; ** not detected.

**Table 3:** The mechanical properties of the synthesized mcl-PUs.

Sample	Tensile Strength (MPa)	Elongation at Break (%)	Young’s Modulus (MPa)
C-P 100/0	14.2 ± 0.9	54.2 ± 9.0	171 ± 14
C-P 80/20	12.3 ± 0.9	59.6 ± 7.3	169 ± 12
C-P 50/50	11.0 ± 1.2	44.0 ± 4.5	128 ± 11
C-P 20/80	6.8 ± 1.2	22.7 ± 3.8	149 ± 6
C-P 0/100	7.4 ± 1.1	8.0 ± 3.3	99 ± 5

## Data Availability

Data supporting this study are openly available from IMAGINE at https://imagine.imgge.bg.ac.rs/handle/123456789/3273 (accessed on 15 May 2026). The LINK will be included upon the acceptance of this manuscript.

## Supplementary Materials

The following supporting information can be downloaded at: https://www.mdpi.com/article/10.3390/polym18121525/s1, Figure S1: NMR spectra of castor oil; Figure S2: NMR spectra of mcl-PHA; Figure S3: (a) FTIR analysis of the mcl-PHA PUs from the 1500 to 2000 cm^−1^ and deconvoluted representative FTIR spectra of the C-P 80/20 sample, (b) The water uptake of the PU films over time; Table S1: The compositional ratio of deconvoluted esters peaks into three peak components at 1740 cm^−1^, free C-O at 1720 cm^−1^ and H-bonded C-O at 1690 cm^−1^ (calculated from FTIR).

## Abbreviations

The following abbreviations are used in this manuscript:

CO	Castor oil
PHA	Polyxydroxyalkanoates
mcl-PHA	Medium-chain-length polyhydroxyalkanoates
HMDI	Hexamethylene diisocyanate
FTIR	Fourier-transform infrared spectroscopy
DSC	Differential scanning calorimetry
TGA	Thermogravimetric analysis
XRD	X-ray diffraction
WCA	Water contact angle
SEM	Scanning electron microscopy
