# Supplementary material for: Sustainable Design of High-Performance Polyurethanes Using Medium-Chain-Length Polyhydroxyalkanoates
Source: Polymers (Basel). 2026 Jun 18;18(12):1525. doi: 10.3390/polym18121525 (PMC13306736; doi:10.3390/polym18121525)
Supplement: Supplementary file 1 [file polymers-18-01525-s001.zip › polymers-4359612-supplementary.pdf]

Supplementary data

a)

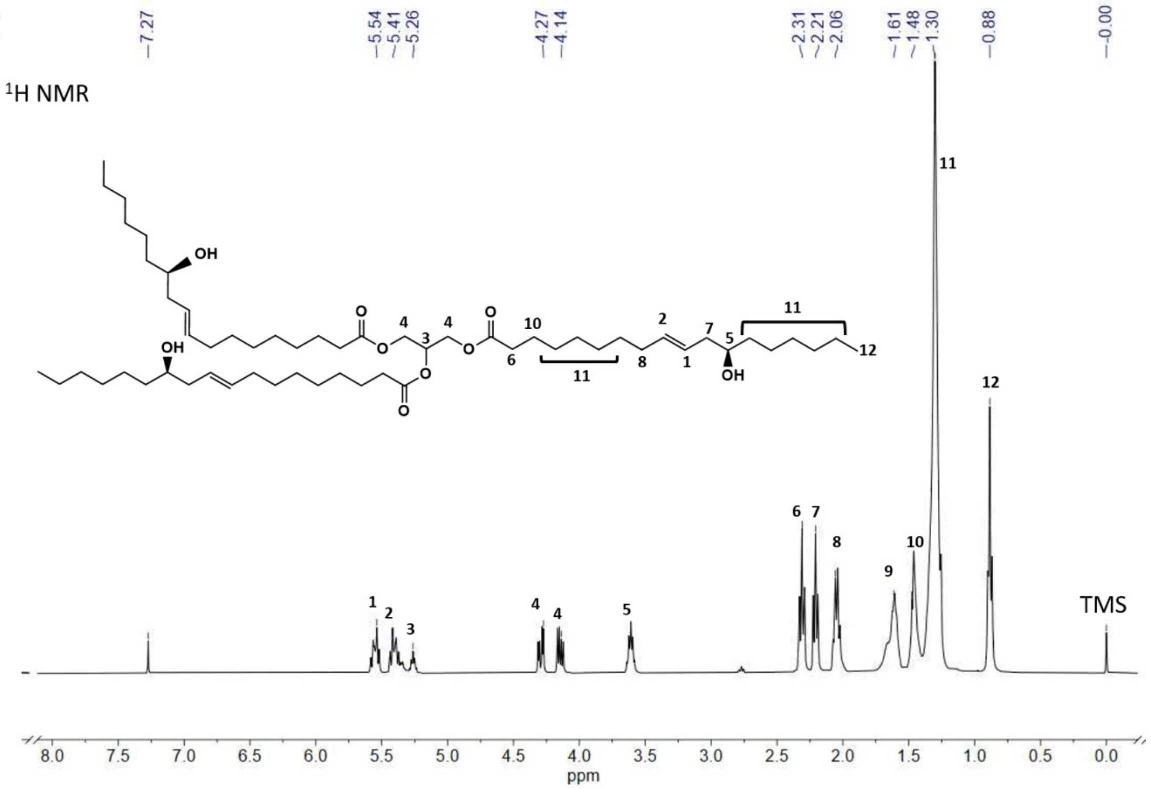

b)

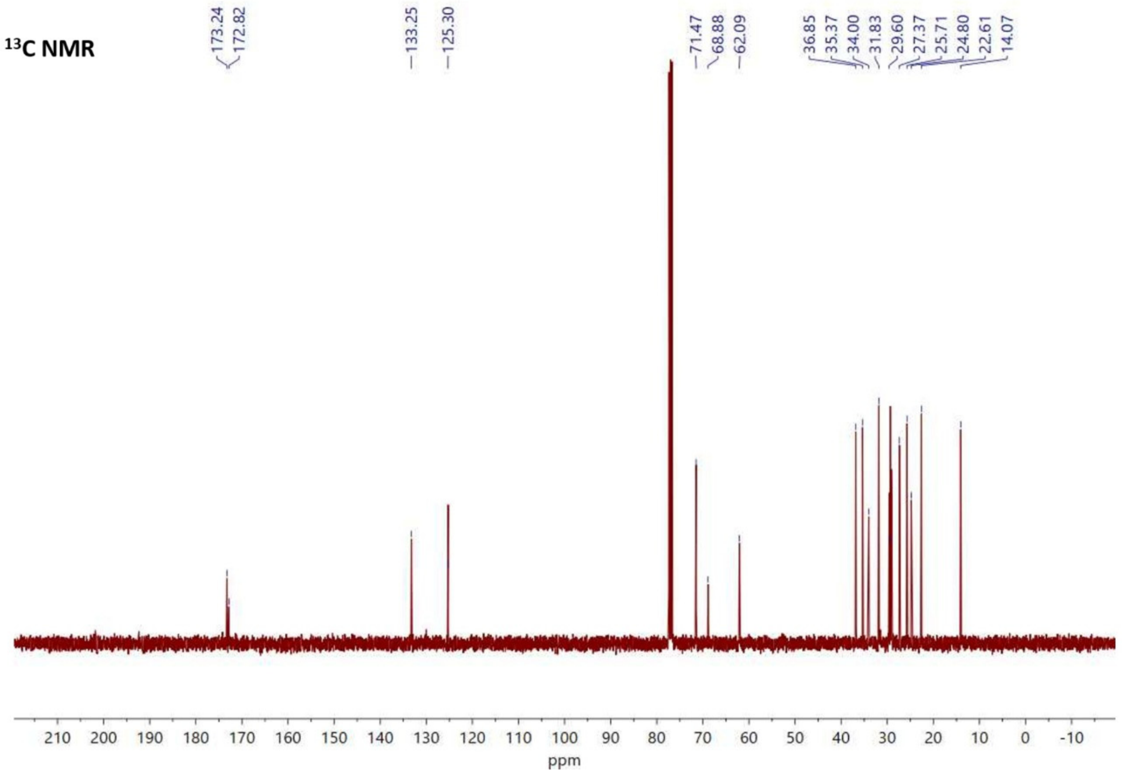

Figure S1. NMR spectra of castor oil.

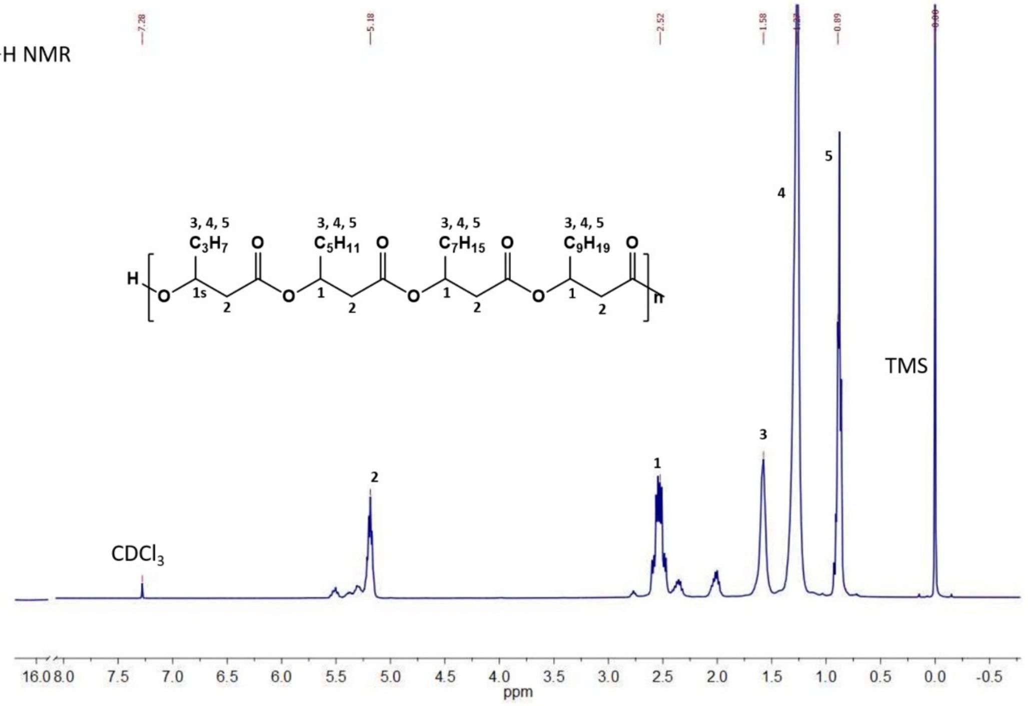

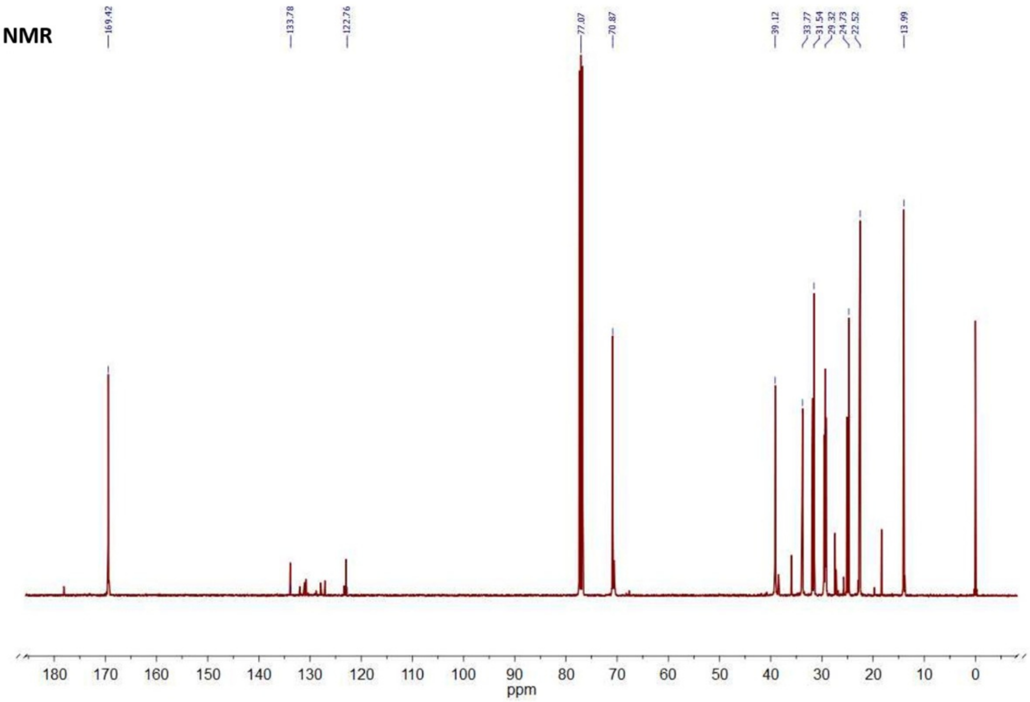

Figure S2. NMR spectra of mcl-PHA.

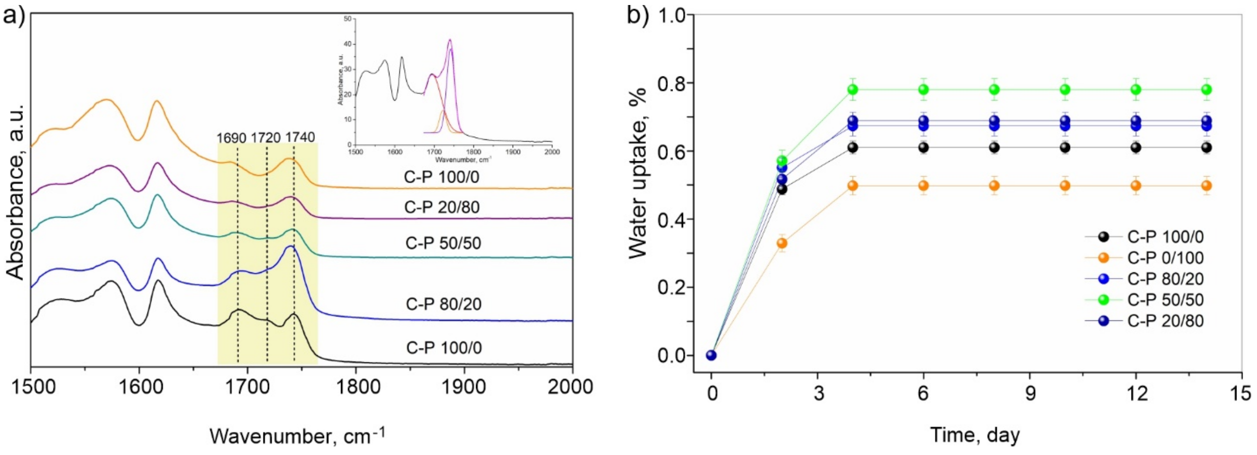

Figure S3. (**a**) FTIR analysis of the mcl-PHA PUs from the 1500 to 2000 cm^{-1} and deconvoluted representative FTIR spectra of the C-P 80/20 sample, (**b**) The water uptake of the PU films over time.

Table S1. The compositional ratio of deconvoluted esters peaks into three peak components at 1740 cm^{-1}, free C—O at 1720 cm^{-1} and H-bonded C—O at 1690 cm^{-1} (calculated from FTIR)

Sample	A_{1740}, %	A_{1720}, %	A_{1690}, %
C-P 100/0	25.2	5.55	69.2
C-P 80/20	35.3	8.10	56.5
C-P 50/50	28.9	7.00	64.1
C-P 20/80	28.4	10.8	60.8
C-P 0/100	24.0	18.5	57.5
